# Patents and the Medical Men

**Published:** 1881-12

**Authors:** 


### EDITORIAL, ETC.

---

*Patents and the Medical Men.*—The *New York Medical Journal* takes the ground in a recent editorial that the clause in the code proclaiming it "derogatory to professional character * * * for a physician to hold a patent for any surgical instrument or invention," should be abrogated as a matter of propriety as well as justice. The difference between the taking out of a patent and vending a secret nostrum is clearly made, and the writer shows that the code should denounce a copyright as well as a patent, the difference between the two being unessential. "The declaration in the code that sets down a patent as derogatory is a dead letter, and should be expunged."

In the dental profession, there are many patents, yet there are not a few who seem to think it not "professional" to patent a good invention. Just why a dentist inventing something valuable to those who will use it if not patented, should not be paid for his invention, when the profession at large are paying handsomely those who *manufacture* the things they are constantly using, is hard to say. It is certainly as fair for one as for the other. As far as the idea of "giving it out to the world" is concerned, the *patient* after all is probably not advantaged, and he is the one who is most largely interested. It is very doubtful if any dentist, or physician would make his fee less because he used an unpatented apparatus or material.

